# Side-hop test can detect deficits in knee functional ability in male athletes following anterior cruciate ligament reconstruction compared to a control group during a battery test performance

**DOI:** 10.3389/fspor.2025.1545226

**Published:** 2025-06-25

**Authors:** Claudio Legnani, Martina Faraldi, Matteo Del Re, Giuseppe Peretti, Alberto Ventura

**Affiliations:** ^1^ IRCCS Istituto Ortopedico Galeazzi, Sport Traumatology and Minimally Invasive Surgery Center, Milan, Italy; ^2^ IRCCS Istituto Ortopedico Galeazzi, Laboratory of Experimental Biochemistry and Advanced Diagnostics, Milan, Italy; ^3^ IRCCS Istituto Ortopedico Galeazzi, E.U.O.R.R. Unit, Milan, Italy; ^4^ Department of Biomedical Sciences for Health, University of Milan, Milan, Italy

**Keywords:** anterior cruciate ligament, ACL reconstruction, activity level, vertical jump, side-hop test, test battery

## Abstract

**Objectives:**

The purpose of this study was to assess whether a battery of jump tests can distinguish between anterior cruciate ligament (ACL) reconstructed patients and control subjects, and to investigate which tests can detect differences in jumping performance between the two groups.

**Methods:**

30 male athletes aged 18 to 50 years matched for sex, age and activity level to a control group of 30 healthy individuals were examined one year after primary ACL reconstruction. Jumping ability was instrumentally assessed by an infrared optical acquisition system using a battery of jump tests including mono- and bipodalic vertical jumps, and a side-hop test. Differences in activity level and jump performance between ACL patients and healthy subjects have been assessed.

**Results:**

The limb used in jump test significantly influenced counter-movement jump (effect size = 0.0145, *p* = 0.0002), drop-jump (effect size = 0.0279, *p* < 0.0001), and side-hop performance (effect size = 0.0029, *p* = 0.002), showing the highest performance for dominant limb on non-dominant limb in healthy subjects, and for uninjured limb on ACL reconstructed limb in ACL-reconstructed patients, in all monopodalic tests. The effect of the intervention was significant only for side-hop test (effect size = 0.1200, *p* = 0.002), with ACL-reconstructed limb and uninjured limb in ACL-reconstructed patients showing a lower side-hop performance compared to non-dominant limb (*p* = 0.014) and dominant limb (*p* = 0.009), respectively.

**Conclusions:**

The capacity to perform side-hop tests was significantly affected in male athletes who had undergone ACL reconstruction compared to control group one year after surgery. Side-hop test can help detecting functional deficits following ACL surgery, thus contributing to estimate athletes' lower limb recovery capacity.

## Introduction

Anterior cruciate ligament (ACL) reconstruction aims to restore pre-injury neuromuscular function and to allow return to sports in patients who had undergone ACL rupture (1, 2).

However, only a percentage of athletes who underwent ACL reconstruction ranging between 44% and 63% is able to resume sports participation at the same pre-injury level (3), with a significant risk of re-rupture especially in young sportsmen (4, 5).

In fact, individuals returning to high-impact activities without optimal neuromuscular control may place themselves at higher risk of reinjury (6). Therefore, before sports resumption, it is crucial to make sure that athletes' knee function has been properly recovered (7–9).

Evidence shows that jump battery tests, which measure knee extensor strength, explosive power, and coordination, are related with rehabilitation outcomes (10–13), and could provide reliable information allowing safe return to sports participation for physicians making decision about return to play (14).

In particular, an important indicator of athleticism in patients performing cutting and pivoting sports, is power generation during vertical jump (15, 16), which demonstrated to be the most reliable and sensitive jump test for determining if normal function had returned following ACL reconstruction (17).

The ability to perform vertical jump has been introduced to evaluate an athlete's functional ability by testing their power and control of movement in scenarios mimicking the physical demands of competition. Following a rehabilitation procedure, jump tests have been utilized separately or in combination in an attempt to determine when the patient should return to recreational practice. Studies demonstrated that between-limbs asymmetries were observed in patients who had undergone ACL reconstruction compared to healthy individuals (18). Although these tests are widely used in many rehabilitation programs, there is disagreement about their reliability in predicting whether passing them lowers the chance of re-rupture, allowing safe return to sport (19).

The purpose of this study was to assess whether a battery of jump tests can distinguish between ACL reconstructed patients and control subjects, and to investigate which tests can detect differences in jumping performance between the two groups.

## Patients and methods

### Patients recruitment

The study group comprised 30 male athletes aged 18 to 50 years who underwent primary ACL reconstruction from January to December 2021, who met the inclusion criteria and signed informed consent, and whose data were retrieved from a parent study (18). IRCCS San Raffaele Hospital's Ethic Committee, Milan, Italy approved this study (IRB number: 57/INT/2020, released from IRCCS San Raffaele Hospital, Milan, Italy).

Inclusion criteria were: Age 18–50 years; male sex; sport participation at recreational/agonistic level. Patients were excluded if they had previous ligamental knee surgery (including the contralateral knee); concomitant ligament reconstructive surgery or treatment for chondral pathology. A control group of 30 healthy, active subjects without history of knee pathology, musculoskeletal and neurological disorders, matched for sex, age, and activity level according to Tegner scale was selected.

### Surgical technique and rehabilitation protocol

All patients underwent arthroscopic assisted ACL reconstruction using doubled autologous hamstring graft (6). Tibial tunnel was drilled using a 55° guide (Acufex; Smith & Nephew, Andover, USA) using as reference the posterior cruciate ligament, while the femoral half-tunnel was prepared either through the medial portal. Fixation was achieved proximally with a cortical suspension device (TightRope; Arthrex Inc., Naples, USA) and distally through a bioadsorbable interference screw (Milagro; DePuy Mitek, Raynham, USA). A brace-free rehabilitation protocol starting the day after surgery was adopted in all patients, with immediate regaining of extension, isometric exercises and walking with crutches with partial weight bearing for the first 3 weeks. Swimming and indoor cycling were allowed after 12 weeks, while after 5 months a protocol of jump technique training and plyometric exercises was started. Surgeons and physiotherapists strictly followed all patients during outpatient and inpatient rehabilitation to monitor progresses and adherence to the protocol.

### Patients assessment

Tegner activity level was administered to all patients 1 year after surgery and to healthy subjects at time of the evaluation. Following a methodology previously described (16), a series of jump tests were performed using an infrared optical acquisition system (OptoGait; Microgate, Bolzano, Italy) 12 months after surgery for the study group. Test data of all participants were completely recorded using Optogait PC Software Version 1.12.0.

A 10 min, self-determined warm-up run on a treadmill was performed by participants to familiarize with the experimental protocol before starting recording data.

The test battery involved bipodalic squat jump (SJ), bipodalic countermovement jump (CMJ), monopodalic CMJ, drop jump (DJ), and monopodalic side-hop test performed with the uninjured limb first, followed by the injured.

Each functional test was executed three times with the exception of the side-hop test which was performed once for each limb. During the test, participants wore tight wear and sport shoes.

Test results were measured in terms of flight duration (milliseconds) and distance (centimeters), and the average of all completed trials was used to compute the score. The percentage of test performance on the unaffected limb during monopodalic jumps relative to the healthy limb represented the Limb Symmetry Index (LSI).

### Statistical analysis

Data were analyzed using Graphpad Prism v8.0 (Prism Software, La Jolla, CA, USA). Data distribution analysis was performed using the D'Agostino Pearson test. Comparison of continuous variables was performed using non parametric Mann-Withney test, comparison of categorical variables was performed using Fisher' Test.

The analysis of monopodalic CMJ, DJ, and side hop has been performed using Two-Way RM ANOVA; *post-hoc* analysis between dominant and non-dominant limbs in healthy subjects, between ACL reconstructed limb and contralateral limb between dominant limb of healthy individuals and contralateral limb of ACL reconstructed patients, and between non-dominant limb of healthy individuals and affected limb of ACL reconstructed patients has been performed using Sidak's multiple comparisons test. The effect size was calculated for each statistical test (rank-biserial correlation for Mann–Whitney test; *η*² for Two-way RM ANOVA). Correlation analyses have been performed using Spearman correlation test. Statistical significance was set at an alpha level of 0.05.

## Results

Patients' demographics and anthropometric data are reported in Table 1. ACL reconstructed patients had undergone surgery an average of 12.6 (S.D.:0.1) months earlier.

**Table 1:** Patient demographics and anthropometric data.

Population characteristics	ACL reconstruced (*n* = 30)	Control group (*n* = 30)	*p*-value
No. of patients	30	30	
Sex			
Male	30	30	
Female	0	0	
Mean age at surgery (±SD and median IQR) (year)	33.2 ± 7.8	33.5 ± 10.9	0.939
	31.9 (26.7–41.3)	32.5 (23.0–44.3)	
Mean BMI (±SD and median IQR) (Kg/m^2^)	27.4 ± 5.1	25.15 ± 2.82	0.035
	27.4 (23.1–30.5)	25.4 (23.1–27.5)	
Dominant/healthy limb (absolute value)			
Right	23	14	0.033
Left	7	6	

SD, standard deviation; BMI, body mass index; ACL, anterior cruciate ligament.

### Tegner activity level

The difference in Tegner score between healthy individuals and patients who had undergone ACL surgery was evaluated. No differences in Tegner scores have been observed between the two groups (effect size = 0.0156, *p* = 0.948), as shown in Table 2.

**Table 2:** Functional recovery and jump test performance indicator (CMJ, CMJ LSI, DJ, DJ LSI, side-hop, and side-hop LSI) in ACL reconstructed patients and healthy individuals.

Jump test performance indicator	ACL reconstructed (30)		Control group (30)		Effect size	*p*-value
Tegner activity level	6.53 ± 1.55 6.00 (5.00–8.00)		6.47 ± 1.41 6.00 (5.75–7.00)		0.0156	0.948
Bipodalic jump						
Bipodalic CMJ (cm)	20.20 ± 4.43 20.35 (18.63–23.68)		24.97 ± 6.53 22.40 (20.18–32.45)		0.3644	0.015
Bipodalic SJ (cm)	21.86 ± 4.66 21.85 (19.60–24.85)		24.06 ± 5.81 22.50 (19.28–30.50)		0.1178	0.438
Monopodalic jump						
	Uninjured limb	ACL reconstructed limb	Dominant	Non dominant		
Monopodalic CMJ (cm)	12.41 ± 3.62 12.50 (10.85–14.90)	11.49 ± 3.53 11.90 (10.30–13.88)	12.73 ± 3.48 12.55 (10.53–16.03)	11.97 ± 3.42 11.00 (8.92–15.13)	*η*^2^group = 0.0033 η^2^limb = 0.0145 η^2^interaction = 0.0001	0.651 0.0002 0.720
Monopodalic CMJ LSI	0.92 ± 0.11 0.91 (0.83–0.99)		0.95 ± 0.15 1.00 (0.80–1.03)		0.0967	0.523
Monopodalic DJ (cm)	14.65 ± 5.17 14.70 (11.60–17.83)	12.60 ± 4.18 14.20 (10.25–16.13)	14.66 ± 5.22 13.85 (10.75–18.00)	13.17 ± 4.49 11.60 (10.25–16.50)	η^2^group = 0.0001 η^2^limb = 0.0279 η^2^interaction = 0.0001	0.929 <0.0001 0.757
Monopodalic DJ LSI	0.89 ± 0.13 0.92 (0.81–0.97)		0.92 ± 0.24 0.90 (0.80–1.10)		0.0711	0.639
Monopodalic side hop (n° of jump/30 s)	40.87 ± 13.75 38.00 (29.00–55.25)	39.40 ± 13.65 37.00 (28.00–52.75)	52.57 ± 16.74 50.00 (41.75–59.25)	50.50 ± 18.08 48.00 (38.75–62.00)	η^2^group = 0.1200 η^2^limb = 0.0029 η^2^interaction = 0.0001	0.006 0.002 0.589
Monopodalic side-hop LSI	0.96 ± 0.07 0.97 (0.93–1.02)		0.95 ± 0.12 0.90 (0.90–1.00)		0.1511	0.315

Data are shown as mean ± standard deviation and median and interquartile range. Comparison of tegner, bipodalic SJ, bipodalic CMJ, and CMJ, DJ, side-hop LSI between the two groups have been performe using Mann–Whiyney test; analysis of monopodalic CMJ, DJ, and side-hop between the two group have been performed using Tw-way RM ANOVA. The effect size and *p*-value is shown for each test. *P*-value was significant when <0.005. Significant *p*-value are shown in bold.

ACL, anterior cruciate ligament; CMJ, countermovement jump; SJ, squat jump; DJ, drop jump; LSI, limb symmetry index.

### Jump test battery

Of the 30 patients who had undergone ACL surgery, 14 had right knee ACL reconstruction, while in 16 cases the operated limb was the left; in the control group the dominant limb was right in 23 on 30 individuals (79%) (*p* = 0.030) (Table 1).

Bipodalic SJ (21.86 cm, SD: 4.66) and CMJ (20.20 cm, SD: 4.43) heights were lower in ACL reconstructed patients compared to healthy individuals (SJ: 24.06 cm, SD: 5.81, and CMJ: 24.97 cm, SD: 6.53) (Table 2). However, the overall results for bipodalic jump have shown significant differences only for CMJ, with a moderate-size difference between the two groups (effect size = 0.3644, *p* = 0.015), as shown Table 2.

**Figure 1:**
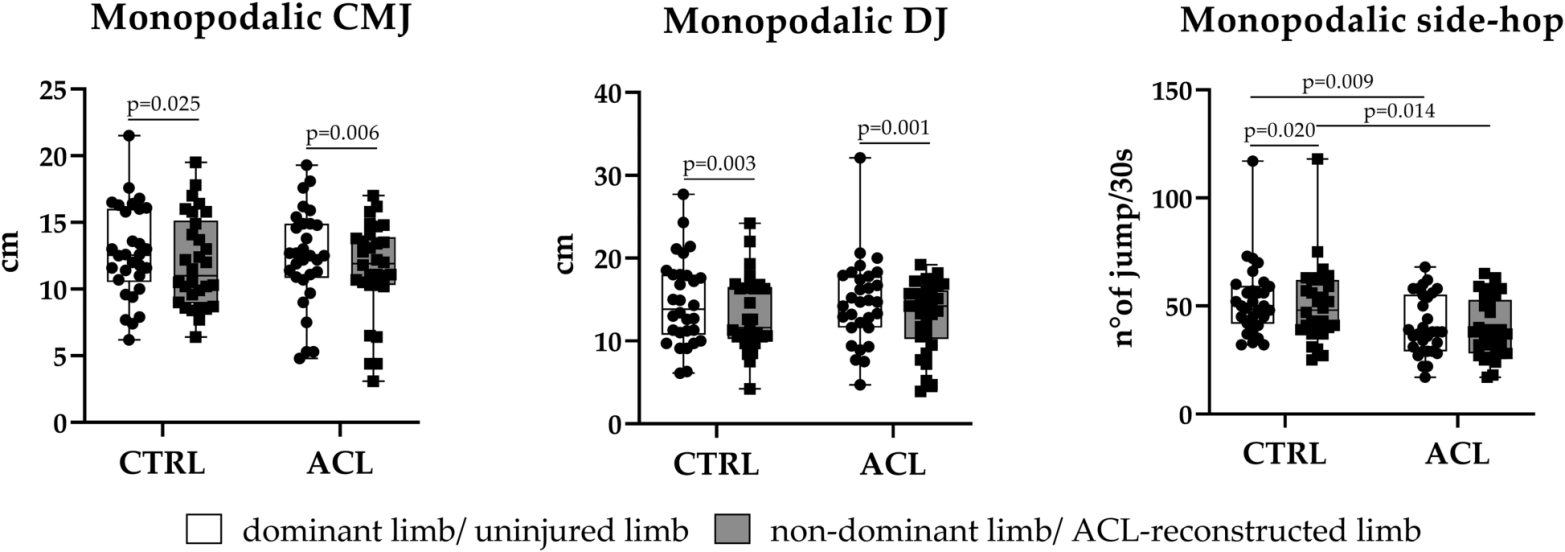
Box-plot showing differences in monopodalic jumping performance (CMJ, DJ, and side-hop) between patients who had undergone ACL surgery and healthy individuals. The black line inside the box represents median value. The lowest bar represents the minimum value, the bottom and top of the boxes represent the interquartile range (25th and 75th percentiles), and the top bar represents the maximum value. Single value data distribution is shown. Data were analyzed by Two-way RM ANOVA; *post-hoc* analysis to compare the jumping performance of dominant and non dominant limb of healthy individual with the jumping performance of ACL reconstructed limb and uninjured limb of ACL reconstructed patients was performed using Sidak's multiple comparisons test. Comparison were significant for *p*-value < 0.05. *P*-value of *post-hoc* analysis was shown.

The limb used in all monopodalic jump tests significantly influence CMJ (effect size = 0.0145, *p* = 0.0002), DJ (effect size = 0.0279, *p* < 0.0001), and side-hop test (effect size = 0.0029, *p* = 0.002) (Table 2). *post-hoc* analysis has highlighted significant differences for CMJ, DJ, and side-hop test between dominant and non-dominant limb of healthy participants (CMJ *p* = 0.024, DJ *p* = 0.003, side-hop *p* = 0.021), and for CMJ and DJ between the ACL reconstructed limb and uninjured limb of ACL reconstructed patients (CMJ *p* = 0.006, DJ *p* = 0.001) (Figure 1).

The effect of the intervention (ACL recontruction) account for a significant portion of variability only for side-hop test (effect size = 0.1200, *p* = 0.006), with ACL reconstructed patients showing the lowest side-hop performance (Table 2). *post-hoc* analysis has shown significant differences between the two group for side-hop test (Figure 1). Lower side-hop performance have been observed in ACL reconstructed limb compared to non-dominant limb of healthy individuals (*p* = 0.014) (Figure 1).

No significant differences have been observed for monopodalic CMJ and DJ between non-dominant limb of healthy individuals compared to ACL reconstructed limb in the study group (*p* = 0.840 and *p* = 0.982, respectively) (Figure 1).

Similarly, lower jump performances have been observed for monopodalic side-hop test between dominant limb of healthy individuals vs. uninjured limb of ACL reconstructed patients (*p* = 0.0091), and not for monopodalic CMJ and DJ (*p* = 0.9213 and *p* > 0.9999, respectively) (Figure 1).

No significant interaction between the main factors (group and limb) have been oberved for CMJ, DJ, and side-hop test (Table 2), suggesting that the effect related to the limb used in each jump test remain consistent across groups. CMJ LSI, DJ LSI, and side-hop LSI have not shown any differences between the two groups (Table 2).

### Correlation analysis

**Figure 2:**
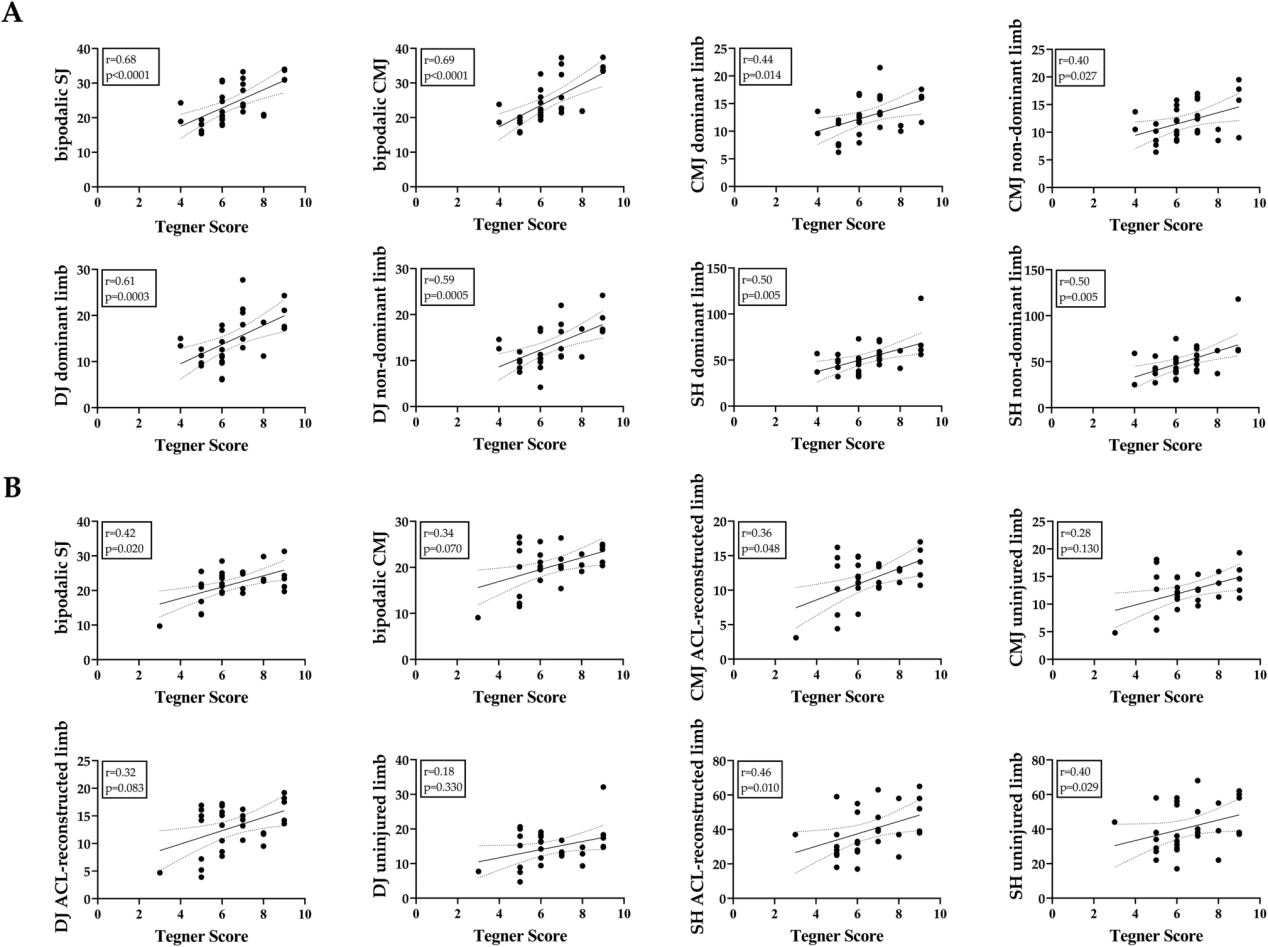
Correlation between functional recovery indicator (Tegner score) and jumping performance (bipodalic sj and cmj, and monopodalic cmj, dj, and side-hop) for **(A)** healthy individuals and **(B)** patients who undergone ACL surgery. Regression line and 95% CI were shown in the graphs. The value of correlation coefficient (r) and *p*-value are also indicated. Correlation were performed using Spearman correlation test and were significant for *p*-value < 0.05.

Correlation analysis have been performed to highlight the association of functional recovery indicator (e.g., Tegner activity level) and jump test performance (e.g., CMJ, DJ, and side-hop test). Considering the group of healthy individuals, both bipodalic patameters (SJ and CMJ) and monopodalic parameters of both dominant and non-dominant limb showed a positive moderate correlation with Tegner score (*p* < 0.05), as shown in Figure 2A.

Considering ACL reconstructed patients, positive weak correlation with Tegner score have been observed for CMJ in ACL reconstructed limb (*p* = 0.048), and side-hop test in both ACL reconstructed limb and uninjured limb of ACL patients (*p* = 0.010 and *p* = 0.029, respectively) (Figure 2B). The highest association with functional recovery (according to Tegner score) in ACL reconstructed patients was observed for side-hop test of ACL reconstructed limb (*r*= 0.46).

## Discussion

Several functional test batteries have been designed to assess return to sport following ACL reconstruction in addition to widely adopted and validated PROMs (20). According to previous researches, vertical jump can reliably measure quadriceps explosive power, strength, and lower limbs neuromuscular control (10–13). In the present study, jump tests were used to distinguish ACL-reconstructed patients from healthy controls, thus evaluating the level of functional recovery following ACL surgery.

Still no consensus exist about which battery of tests may provide the most accurate evaluation of performance capacity, giving an esteem of timing to return to sport. In addition, test batteries performed in the clinical setting need to be cost-effective and simple to reproduce.

According to Kotsifaki et al, ACL reconstructed athletes reported deficits while performing vertical jumps. Authors suggest that vertical jumps may be reliable in detecting functional deficit in patients cleared to return to sport after ACL reconstruction (10).

In the current study, the ability to perform bipodalic jump in patients who had undergone ACL reconstruction was significantly lower compared to healthy subjects while performing CMJ, but not while performing SJ.

Concerning monopodalic jump tests, according to our findings, ACL reconstruction significantly affected the side-hop test, as the performance of ACL reconstructed patients differed from healty subjects only while performing this task. Indeed, jump performance in reconstructed limbs in patients who had undergone ACL surgery was significantly lower compared to the non-dominand side of healthy subjects while performing side-hop test, but not while executing CMJ and DJ.

Reduced jump performance while performing side-hop tests may provide insight as to why individuals undergoing ACL surgery exhibit decreased lower limb function; in fact, one of the main mechanisms for ACL injuries during cutting and pivoting sports has been identified as change of direction, occurring during side-stepping maneuvers (21). This persistent deficit in high-demanding functional tasks may be a reason for unsuccessful surgery and higher re-injury rates in subject returning to sports requiring sudden change of direction (22).

Our findings support the knowledge that adding muscle strength training to rehabilitation can improve athletic performance of ACL reconstructed sportsmen (23).

The 30 s side-hop test, which tests knee stability in the frontal plane and causes muscular fatigue, may be used in both clinical and research settings with both healthy and injured people, is one of the helpful functional performance tests to evaluate lower limb function in a secure medical environment. According to our results, the side-hop test is able to detect lower limb asymmetries one year after surgery in ACL-recostructed male athletes. Conversely, Hamrin Senorski et al. reported a low correlation between return to performance and the ability to perform side-hop test only in female patients (24).

Pre-operative counseling should consider the possibility that return to sport within 12 months following ACL surgery may not be a feasible objective for all patients undergoing ACL reconstruction. Also, the persistence of functional deficits during high-demanding tasks may suggest to add extralateral procedures to ACL reconstruction in order to improve rotational stability and ultimately to enhance the ability to perform side-stepping maneuvers (25).

Interestingly, increased jump performances have been reported concerning dominant limb of healthy individuals compared to the uninjured limb of ACL reconstructed patients concerning monopodalic side-hop test. This finding may suggest that following ACL reconstruction, an impairment concerning both limbs could be observed according to the result of a side-hop test.

No effects of ACL reconstruction have been observed concerning monopodalic CMJ and DJ. This result toghether with the lack of a statistically significant difference between the two groups while performing CMJ and DJ tests, makes to appear questionable the role of these tests to discriminate between ACL reconstructed patients and healthy subjects.

Limitations of the present study include its research design and the relatively small sample size, thus limiting the ability to detect small differences between groups regarding some parameters. In our study, only male patients who underwent ACL surgery with doubled autologous hamstrings were considered for inclusion, therefore our data may not be generalizable to females or to athletes who had undergone ACL reconstruction with other other surgical techniques. Patients were not matched for BMI, which tended to be higher in ACL reconstructed patients, and this constitutes a further study limitation. OptoGait was chosen as a simple and low-cost instrument, which can be easily used in the clinical setting and allows to perform reliable measurements of functional ability. Its validity concerning the evaluation of spatiotemporal gait parameters has been previously reported (26). We acknowledge that many variables influence jumping performance, and the use of jump height as an expression of neuromuscular restoration following ACL reconstruction represents a limit of the present study.

Future studies with larger cohorts, different populations, different tests and surgical techniques are required to investigate the correlation between the variables affecting jumping performance to provide new reliable data for investigating knee recovery following ACL reconstruction.

## Conclusions

Reduced jump performance while performing side-hop tests were observed in ACL reconstructed patients one year after surgery compared to a control group of healthy volunteers. Since the significant effect of ACL reconstruction was observed only on side-hop performance, side-hop test can help detecting functional deficits following ACL surgery, thus contributing to estimate athletes' lower limb recovery capacity.

## Data Availability

The datasets presented in this study can be provided by the authors upon reasonable requests.
